# Human-centered visualization technologies for patient monitoring are the future: a narrative review

**DOI:** 10.1186/s13054-023-04544-0

**Published:** 2023-06-28

**Authors:** Greta Gasciauskaite, Justyna Lunkiewicz, Tadzio R. Roche, Donat R. Spahn, Christoph B. Nöthiger, David W. Tscholl

**Affiliations:** Institute of Anesthesiology, University Hospital Zurich, Raemistrasse 100, 8091 Zurich, Switzerland

**Keywords:** Visualization technologies, Situation awareness, User-centered design, Patient monitoring, Intensive care unit, Perioperative medicine, Patient safety

## Abstract

Medical technology innovation has improved patient monitoring in perioperative and intensive care medicine and continuous improvement in the technology is now a central focus in this field. Because data density increases with the number of parameters captured by patient-monitoring devices, its interpretation has become more challenging. Therefore, it is necessary to support clinicians in managing information overload while improving their awareness and understanding about the patient’s health status. Patient monitoring has almost exclusively operated on the single-sensor–single-indicator principle—a technology-centered way of presenting data in which specific parameters are measured and displayed individually as separate numbers and waves. An alternative is user-centered medical visualization technology, which integrates multiple pieces of information (e.g., vital signs), derived from multiple sensors into a single indicator—an avatar-based visualization—that is a meaningful representation of the real-world situation. Data are presented as changing shapes, colors, and animation frequencies, which can be perceived, integrated, and interpreted much more efficiently than other formats (e.g., numbers). The beneficial effects of these technologies have been confirmed in computer-based simulation studies; visualization technologies improved clinicians’ situation awareness by helping them effectively perceive and verbalize the underlying medical issue, while improving diagnostic confidence and reducing workload. This review presents an overview of the scientific results and the evidence for the validity of these technologies.

**Supplementary Information:**

The online version contains supplementary material available at 10.1186/s13054-023-04544-0.

## Background

Information technology is now standard in perioperative and intensive care medicine, and has significantly improved patient care [1, 2]. Patient monitoring is a highly recommended standard in the International Standards for a Safe Practice of Anesthesia [3]. Monitoring is also essential in critical care to optimize patient ventilation, hemodynamics, and metabolism [4, 5].

### Data overload

As new sensors are added to patient monitoring and the number of parameters monitored increases, so does the complexity of their interpretation and the cognitive load that clinicians face [6–9]. There is an imbalance between the amount of data available and the ability to identify important information. More data may provide more information for computers to use, but it does not always translate to a corresponding increase in meaningful information for people.

According to cognitive load theory, humans cannot process large amounts of data over extended periods of time because working memory has a limited capacity, typically 5 to 9 items at a time [10, 11]. Overloaded working memory leads to poorer information processing, comprehension, and retention. In addition, care providers face an emotional burden dealing with life-critical situations, which also affects mental acuity [12–15]. The combination of cognitive and emotional load may lead to occupational distress, fatigue, and burnout [16, 17], which can affect quality of care and is associated with reduced patient safety, longer hospitalizations, and even increased mortality [15, 18]. Clinicians require support in managing such highly complex situations.

Adapting the work environment around the skills and needs of care providers could reduce their workload and help them manage critical situations both cognitively and emotionally. One way to improve the way clinicians perceive patient information is to apply user-centered design principles to patient monitoring technology [19–21].

Patient monitoring has almost exclusively used the single-sensor–single-indicator principle, a technology-centered form of information presentation in which individual parameters are measured and displayed as separate numbers and waves [22, 23]. Care providers must process, integrate, and interpret each vital sign individually before they can decode the meaning. This type of model does not utilize the capabilities of human sensory perception or provide optimal awareness of the patient’s current state [23]. However, if multiple parameters derived from multiple sensors are integrated into a single indicator, care providers can assess the full range of vital signs in parallel instead of piecemeal [24]. Computer-based and high-fidelity simulation studies on user-centered design-based systems have shown that patient-monitoring data provided in the form of changing shapes, colors, and animation frequencies, contributed to higher accuracy in clinical diagnosis, quicker decision-making, lowered perceived workload, and increased perceived diagnostic certainty [25–28].

### The psychology and cognitive neuroscience behind user-centered data visualization

Humans perceive and organize what they see in ways to help them understand and assimilate the information [29]. The Gestalt principles of perception—proximity, similarity, enclosure, closure, continuity, and connection—emerged in the early twentieth century and are still recognized as accurate descriptions of human visual behavior. Based on the Gestalt principles, objects are perceived as a group if they are close together, share similar attributes, appear to have a boundary around them, appear to be a continuation of one another, and/or are connected [29–31]. Incorporating these principles in the design of user-centered technologies supports how humans perceive and interpret visual information to make data more intuitive.

Dual-processing theory categorizes human thinking and visual information processing into two complementary types: associative (system 1) and reasoning (system 2) [32–36]. System 1 enables fast, instinctual decision-making without relying on working memory. It is controlled by the visual cortex and operates autonomously and is driven by emotions and intuitive judgments [32, 34–38]. System 2 is associated with slow and rational decision-making, mainly managed by the frontal cortex [32–40]. The two systems work in parallel and are interconnected to form a coherent perception of visual information.

The human brain can almost instantaneously detect color, motion, and shape; integrate it and make associations. These principles can guide the design of user-centered patient monitoring technologies that prioritize awareness and optimize human sensory perception. In contrast, the single-sensor–single-indicator model, which presents information independently, may demand increased cognitive effort.

### Situation awareness

Situation awareness is based on collecting information from multiple sources in the environment, comprehending what the information means, and using it to think ahead about what might happen next [19]. It involves building and sustaining a dynamic awareness of the situation and risks present in an activity. The principles of situation awareness originated in aviation psychology, but are also applied in intensive care medicine [41] and anesthesia [20], where managing dynamic and complex situations is critical. Approximately 80% of treatment errors in the intensive care unit and in the intraoperative setting are due to inadequate situation awareness of health care providers [42, 43].

User-centered design is used as a framework for developing situation awareness-oriented systems [44] to achieve optimal functioning of overall human–computer interaction and to ensure patient safety [19, 44]. Systems designed with situation awareness in mind provide a more comprehensive overview of the patient’s condition than just separate sensor information and can help healthcare providers potential issues quicker.

## User-centered technologies for patient monitoring

Several user-centered technologies for patient monitoring have been designed based on the psychology and cognitive neuroscience principles previously mentioned and the capabilities of human sensory perception. The philosophy behind these technologies is based on user-centered design by Endsley [19], principles of logic from Tractatus Logico-Philosophicus by Wittgenstein [45], and human–computer interaction described in the NASA publication On Organization of Information: Approach and Early Work by Degani et al. [46]. The user-centered design principles suggest using direct visual representations of data to facilitate situation awareness. Wittgenstein’s theory states that a logical picture has a meaningful commonality with the reality it attempts to represent. The NASA publication underlined that the highest level of “order and wholeness” could be achieved by integrating all required data into a single display. Such an approach allows us to immediately see if all parameters are within the normal range, providing instant reassurance and reducing the cognitive load required to read each number individually. The goal of the described technologies is a situation awareness-oriented interface to convey the information the health care providers as quickly as possible and with the lowest cognitive effort [19].

### Visual patient

**Fig. 1:**
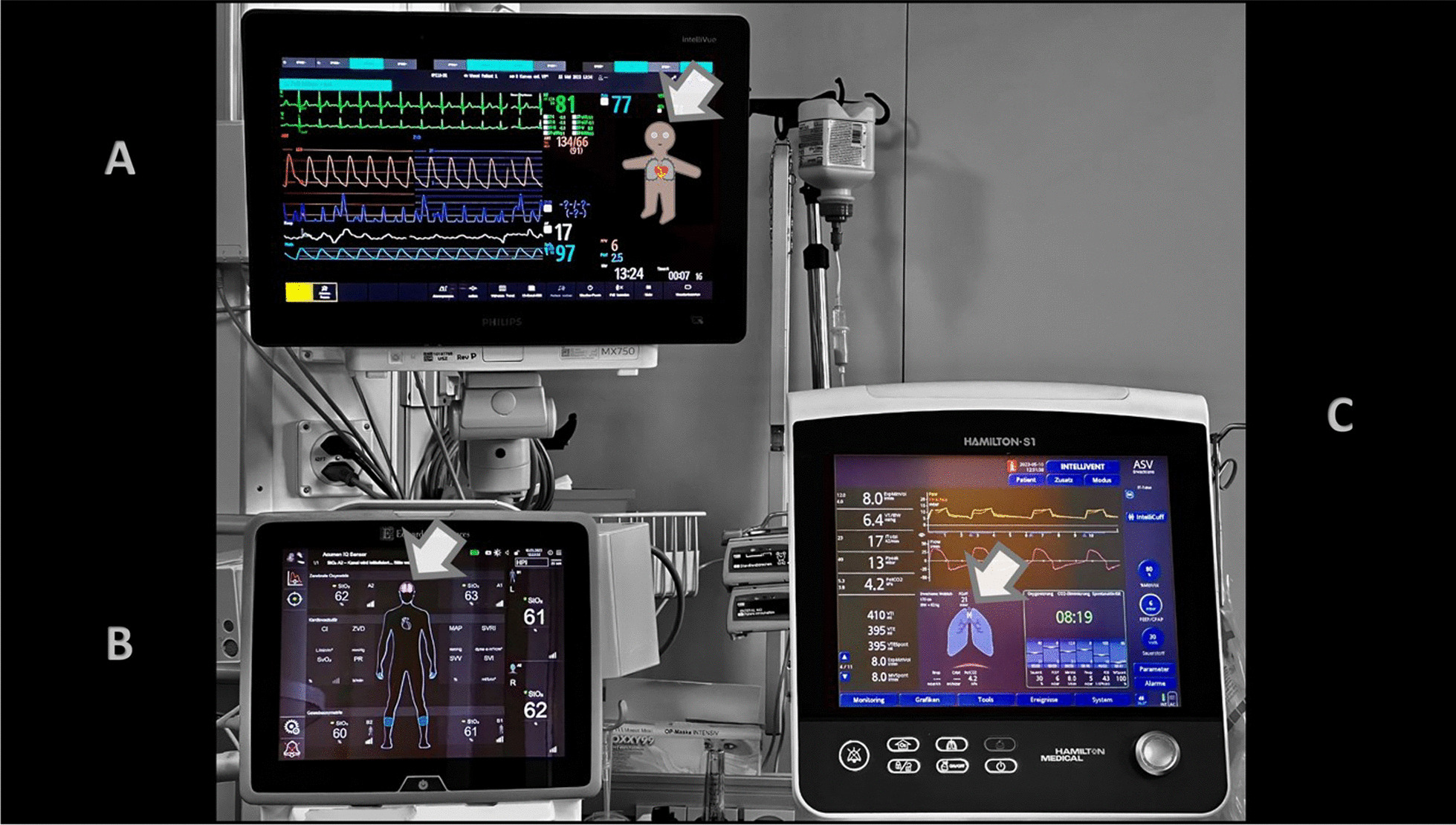
Visual presentation of vital signs in a critical care unit at the University Hospital Zurich. **A** Philips Visual Patient Avatar **B** Edwards Lifesciences Tissue Oxygenation Physiology View **C** Hamilton Medical Dynamic Lung panel

**Fig. 2:**
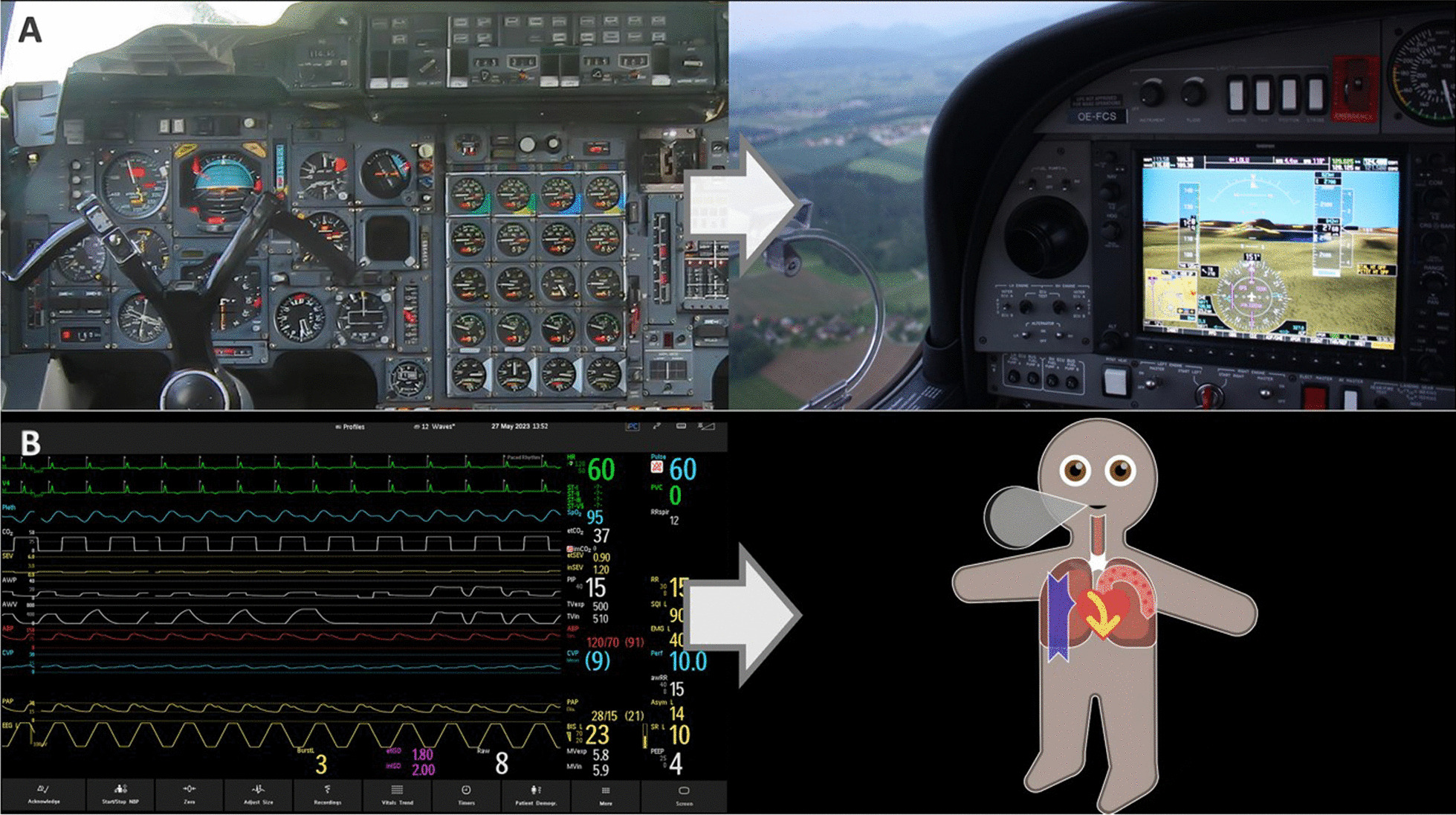
How multiple individual readings can be presented visually. **A** Synthetic Vision converts complex flight data into a digital twin of the view out the windscreen. **B** Visual Patient converts multiple vital signs into an avatar. Visual Patient technology was initially developed at the University Hospital Zurich, Switzerland

**Fig. 3:**
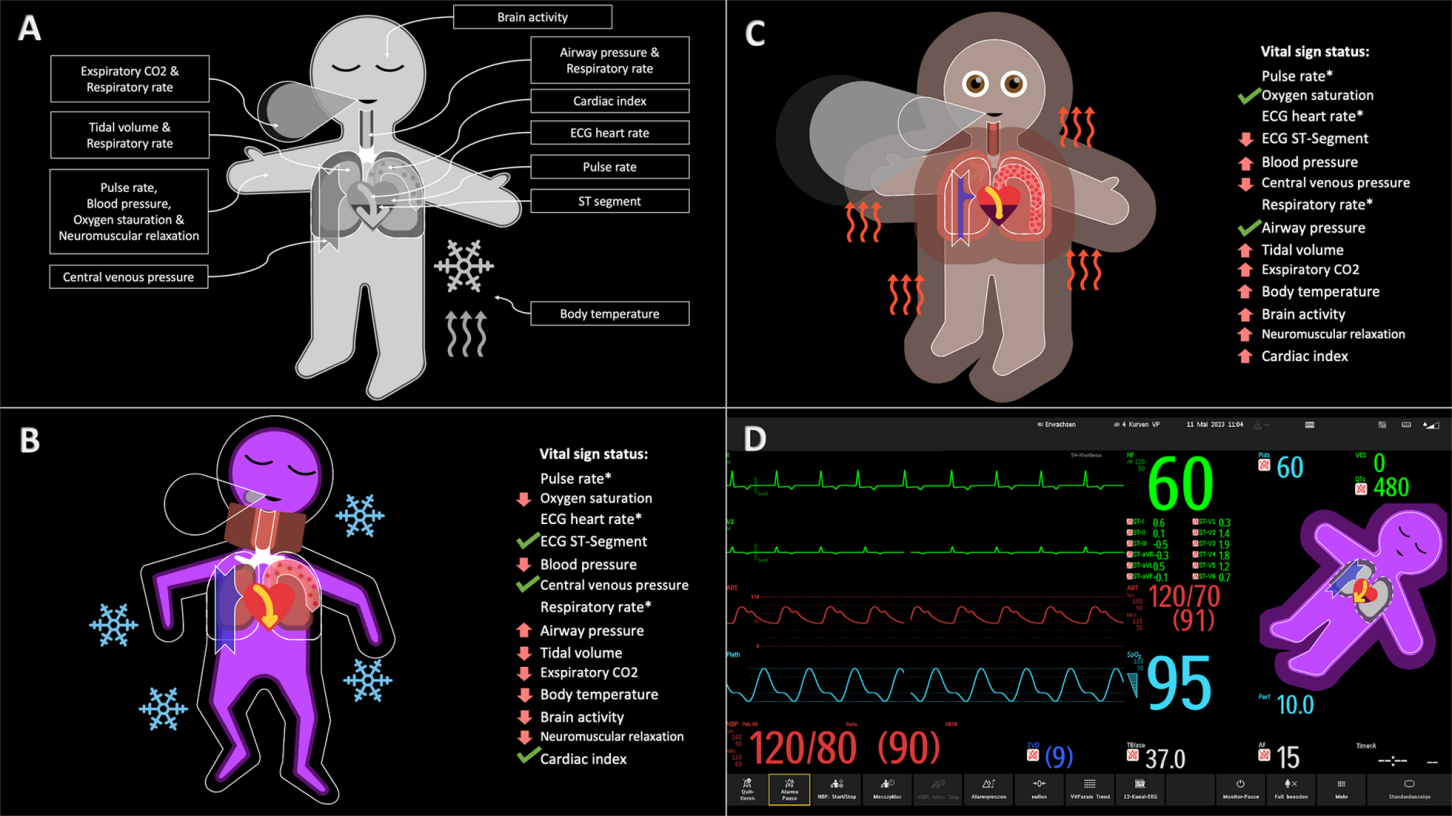
Visual Patient. **A** Vital signs that are visualized by Visual Patient and locations of their appearance. **B, C** Visual Patient showing various vital signs deviations and their verbal explanations.** D** Split-screen, showing a combination of conventional monitoring and Visual Patient

Visual Patient is a situation awareness–oriented visualization technology for patient monitoring developed into the Philips Visual Patient Avatar (Royal Philips, Amsterdam, Netherlands) [21, 47]. Visual Patient (Fig. 1A, 2B, 3) uses shapes, colors, and animation in the form of an avatar to convey information about vital signs.

The Visual Patient system originated from Synthetic Vision (Fig. 2A), a visualization technology used in aviation that combines data from multiple numerical values into a 3D visual representation of the outside world to improve the flight crews’ situation awareness [48, 49]. Aircraft attitude, altitude, airspeed, distance to obstacles, and other data are presented to pilots as if they were looking through a windshield on a clear day, regardless of the outside conditions, which allows for the overall situation to be interpreted quicker and with greater confidence.

The visual data presented in Visual Patient (Fig. 1A, 2B, 3) are modeled after and look like real-life, for instance, the avatar exhales a regular, small, or large cloud of CO_2_, depending on the exhaled CO_2_ concentration measured. The design of the visualizations, i.e., shapes, colors, and animation paces used by Visual Patient was improved and re-validated several times during the initial development process with 150 care providers in two centers [25]. After a 6-min educational video (Additional File 1), all visualizations eventually reached an inter-rater reliability of > 94%. In a study performed with 30 participants, > 70% of the visualizations of the revised design used in the product were intuitively recognizable without any training [50].

In critical situations, problems need to be verbalized for others to take action. In urgent situations, critical information that is not perceived cannot be verbalized [51]. A high-fidelity simulation study conducted with 52 teams showed Visual Patient was associated with a higher probability of verbalization of the emergency cause compared with a standard monitor (hazard ratio [HR] 1.78; 95% CI 1.13–2.81; *p* = 0.012) [52]. In a computer-based study by Tscholl et al. with 32 participants, anesthetists working with Visual Patient perceived almost twice as many vital signs during the same time compared with working with a conventional display (9 vs. 5; *p* < 0.001) [25]. Similar findings were seen in a computer-based study with 50 care providers featuring a simulated intensive care setting [53]. Using a version of Visual Patient, which incorporated patient-inserted devices like central venous line, arterial line, and urinary catheter, resulted in a higher rate of accurate assessment of vital signs and inserted devices compared to a standard monitor (rate ratio [RR] 1.25; 95% CI 1.19–1.31; *p* < 0.001). These findings show that Visual Patient increases perception of the underlying problem, leading to verbalization and, ultimately, increased situation awareness.

Diagnostic confidence is an essential component in clinical decision-making. Increased diagnostic confidence can positively affect situation awareness by reducing stress from uncertainty [25]. In the Tscholl et al. study, with 32 participants, Visual Patient significantly increased anesthesia providers’ diagnostic confidence in patient monitoring compared with current standard monitoring (2 = certain vs. 1 = uncertain; *p* < 0.001 [25]. Bergauer et al. also found staff members in a critical care setting were more confident when interpreting vitals with Visual Patient vs. a conventional display (odds ratio [OR] 3.32; 95% CI 2.15–5.11; *p* < 0.001) [53].

A high cognitive workload can reduce the ability to process data [54]. A computer-based study with 32 participants found that Visual Patient lowered subjectively perceived workload (NASA Task Load Index [NASA-TLX] 60 vs. 76; *p* < 0.001) [25]. In a computer-based study with 38 participants, Visual Patient increased perceptual performance when working under distractions [55]. In this prospective multicenter study, anesthesia providers evaluated 3-s and 10-s scenarios using standard and Visual Patient monitoring while being distracted by a standardized simple calculation task [56]. Anesthesia providers remembered more vital signs under distraction using the avatar in the 3-s scenario: 6 vs. 3; *p* < 0.001, and in the 10-s monitoring task: 6 vs. 4; *p* = 0.028. In this study, participants rated perceived workload lower under distraction with the avatar in the 3-s scenario: 65 vs. 75; *p* = 0.007, and in the 10-s scenario: 68 vs. 75; *p* = 0.019.

Visual Patient may alert clinicians to vital sign changes earlier because it remains visible in their peripheral vision. In a multicenter eye-tracking study in which 38 anesthetists used only their peripheral vision to look at patient-monitoring scenarios, participants using Visual Patient were able to detect significantly more vital sign changes than with conventional monitoring, with the median number of correctly identified vital sign changes rising from 3 to 12 (*p* < 0.001) in the first scenario and from 3 to 8 (*p* < 0.001) in the second [57].

Tscholl et al. in an eye-tracking study with 32 anesthetists showed participants visually fixated on more vital signs for a longer time when using Visual Patient monitoring and that there was a statistically significant association between the visual fixation of a vital sign and its correct perception [24].

Regarding use of Visual Patient, in a qualitative online survey with 38 anesthesiologists, more than 80% of anesthesia personnel found Visual Patient intuitive and easy to learn [27]. In another study with 30 participants, two-thirds stated that the adapted avatar design version used in Visual Patient looked professional for clinical use [58].

### AlertWatch

**Fig. 4:**
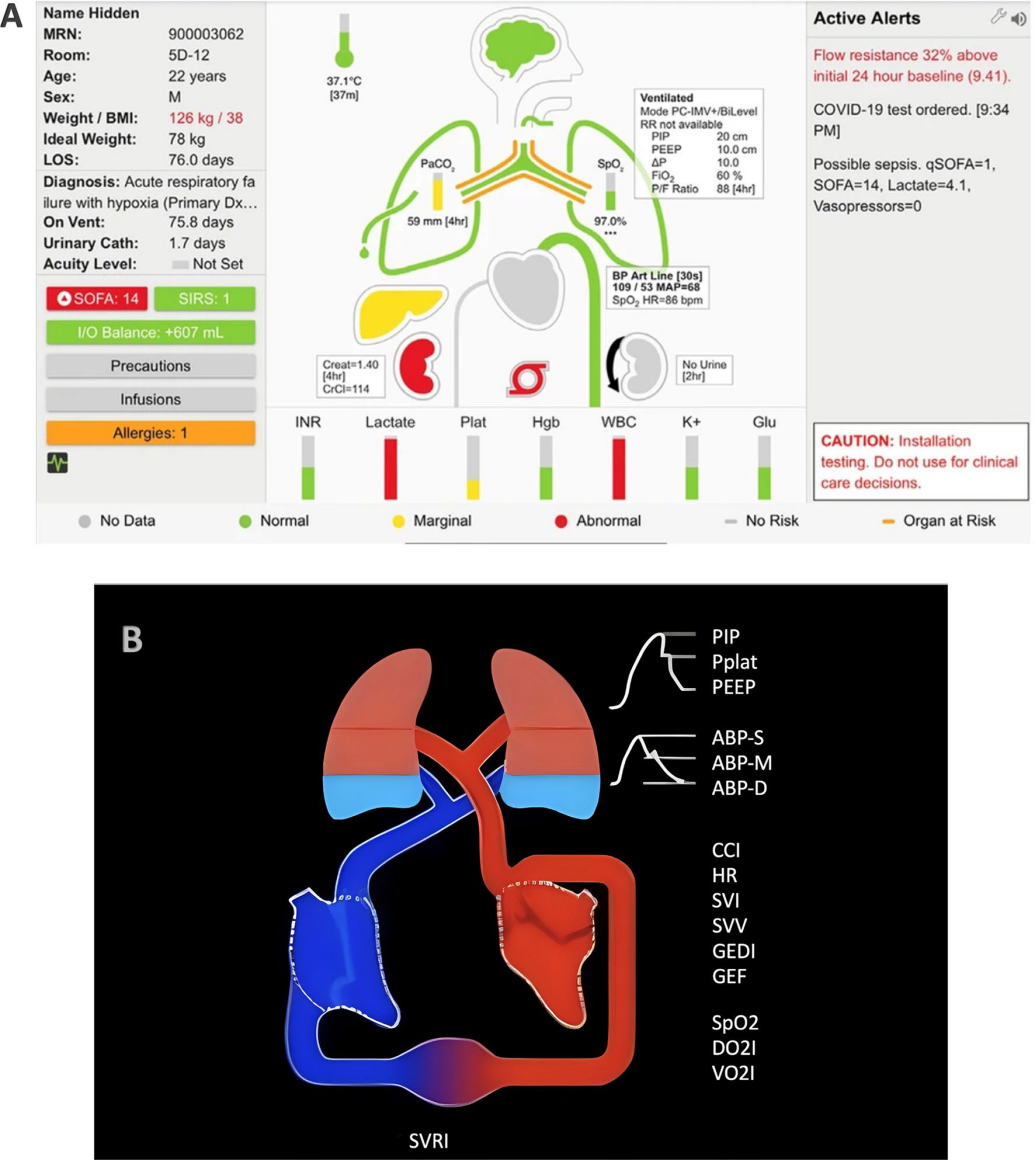
**A** AlertWatch:OR AlertWatch® OR. Accessed on June 5, 2023, at https://doi.org/10.1186/s12871-018-0478-8. **B** Mindray HemoSight. Accessed on June 5, 2023, at https://doi.org/10.1186/s13613-016-0119-7. Both images under Creative Commons Attribution 4.0 International License (http://creativecommons.org/licenses/by/4.0/)

AlertWatch:OR, AlertWatch:OB, Alertwatch:AC (AlertWatch, Inc. Ann Arbor, MI, US) (Fig. 4A) are multifunction decision-supporting systems for intraoperative anesthetic care, obstetric care, and remote monitoring [59]. Information, such as patients’ comorbidities and lab values, are pulled from multiple networked information systems and integrated with live physiologic data in a real-time audiovisual display [60]. Complex information is conveyed through identifiable icons representing organ systems, color-coded to indicate normal (green), marginal, and abnormal (red) ranges. These organ icons are animated based on the patient's actual heart rate, pulse rate, or respiratory rate. The technology also includes demographic data and text alerts.

A retrospective study [61] evaluating 26,769 patients showed that AlertWatch:OR statistically significantly improved multiple process measures, i.e., crystalloid fluid administration (5.88 ml/kg/h; 95% CI 4.18–8.18; vs. 6.17; 95% CI 4.32–8.79; *p* < 0.001), tidal volume greater than 10 ml/kg (28% vs. 37%, *p* < 0.001, adjusted OR 0.65; 95% CI 0.53–0.80; and hypotension 1 vs. 1 min, *p* < 0.001). A further study comparing 791 cases managed with AlertWatch:OR to 1,550 managed without it showed that use of the technology was associated with a significant increase in desirable intraoperative glycemic management (adjusted OR 1.55; 95% CI 1.23–1.95; *p* < 0.001) [62].

A retrospective observational study of 120 deliveries complicated by severely morbid postpartum hemorrhage after delivery showed that AlertWatch:OB identified 10 cases that were not identified by the standard Maternal Early Warning Criteria [63]. An online survey with 273 participants who used the system showed that 83% of the users felt it should remain in place, indicating high user acceptance [64].

### Dynamic lung panel and PulmoSight

The Dynamic Lung panel (Hamilton Medical AG, Bonaduz, Switzerland) visualizes respiratory monitoring data from ventilators in an animated anatomical lung image (Fig. 1C) [59]. The system visualizes multiple parameters in real-time, such as tidal volume, patient triggering, cuff pressure, resistance, compliance, SpO2, and pulse rate. For instance, the size of the lungs changes with each breath to represent tidal volume, while the shape of the lungs indicates compliance. Color coding is employed, with the bronchial tree color reflecting resistance levels. PulmoSight (Mindray Medical International Limited, Shenzhen, Guangdong, China) also uses an anatomical lung image with a bronchial tree and trachea to visualize respiratory parameters [59]. The visualization showcases the patient's breathing initiation, resistance, compliance, and tidal volume by modifying the contours of different lung areas. Thicker tracheal walls depict increased resistance. Use of animation is limited to brightness changes indicating a breath.

Wachter et al. applied an iterative design process to develop a graphical lung visualization that uses some similar visualizations as the Dynamic Lung panel and PulmoSight [65]. A study [66] with 19 participants showed that care providers identified and managed selected simulated pulmonary events more quickly, more accurately, and with reduced perceived workload using this graphical display.

### HemoSight and physiology screen

HemoSight (Mindray Medical International Limited, Shenzhen, Guangdong, China) is a visualization for advanced hemodynamic monitoring (Fig. 4B) [59]. The visualization includes the heart, lungs, blood, and vascular system, with separate venous and arterial legs. The color of the arterial vasculature reflects measured oxygen saturation, while the color of the venous vasculature represents central venous saturation. Blood vessel diameter changes indicate systemic vascular resistance index values. The animation displays the patient's heart rate visually. The size of the heart during systole indicates the ejection fraction, while the size during diastole indicates the end-diastolic volume index. Changes in lung fluid level indicate extravascular lung water index values. The Physiology screen (Edwards Lifesciences Corp., Irvine, CA, US) is also a visualization for advanced hemodynamic monitoring that shows the interaction between the heart, lungs, blood, and vessels [59]. The animation of blood flow adjusts based on cardiac output/index and target ranges (slow, normal, fast). Vessel constriction reflects systemic vascular resistance and the chosen target range. The heart's beating in the physiology view represents the pulse rate, though not the exact beats per minute. Additional visualizations are included for tissue oxygenation measurement. In a comprehensive representation, the patient avatar displays numerical oxygenation values specific to anatomical locations like the cerebral region and hemodynamic parameters (Fig. 1B).

### Alarm status visualizer

Alarm Status Visualizer (Masimo Corp., Irvine, CA, US) shows visual alarm indicators on a three-dimensional anatomical image of a human body that associates alarm status with green (no alarm), yellow or red color [59]. The avatar-based picture includes a brain, heart, lungs, and vascular system that change colors based on vital sign status. The heart and lungs can also be animated according to pulse rate and respiratory rate parameters.

### Emerging technologies

#### Visual clot

**Fig. 5:**
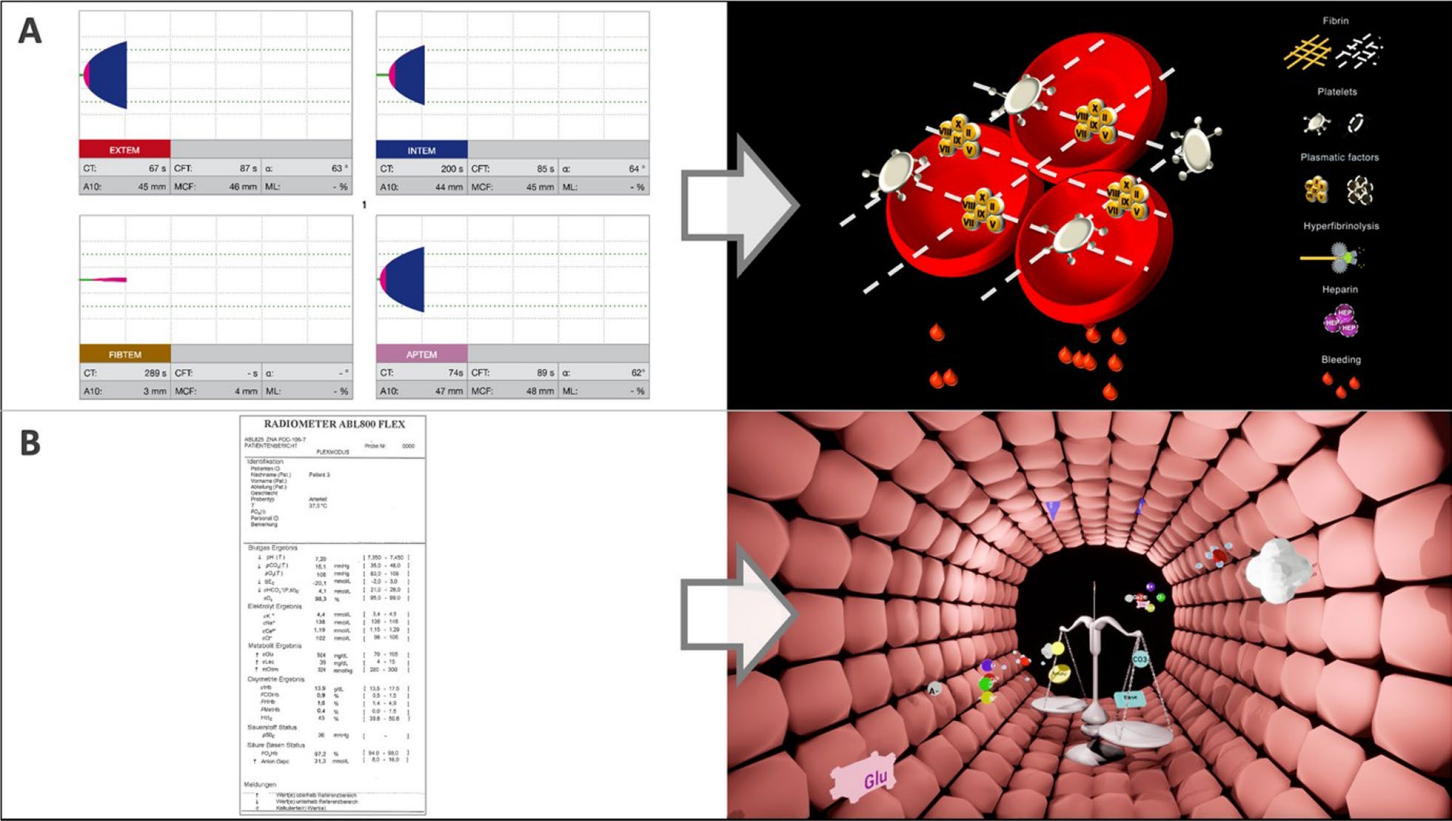
Visualization technologies under development. **A** Left: ROTEM temograms; right: Visual Clot. **B** Left: standard arterial blood gas printout; right: Visual Blood. Visual Clot and Visual Blood are technologies currently under development at the University Hospital Zurich, Switzerland

Interpreting rotational thromboelastometry (ROTEM) results is a complex and cognitively demanding task that requires significant training [67, 68]. Visual Clot is a visual representation of viscoelastic test results under development by our research group (Fig. 5A, Additional file 2: Video) [69, 70]. The technology creates a real-time, 3D-animated model of a blood clot based on thromboelastometry parameters representing the different components of hemostasis, including platelets, plasmatic factors, and fibrin. It can also demonstrate the impact of heparin and hyperfibrinolysis.

A high-fidelity simulation study with 59 teams showed that anesthetists using Visual Clot were 2.2 times more likely to voice the correct therapy (HR 2.27; 95% CI 1.29–3.99; *p* = 0.005) [71]. The anesthetists in the study working with Visual Clot also had a lower median time to administer a first correct targeted coagulation product (269 s vs. 370 s; *p* = 0.003). Generally, physicians presented with the results of viscoelastic tests in the form of Visual Clot, had approximately 56% higher chance of performing the correct therapeutic measures (RR 1.56; 95% CI 1.0–2.47; *p* = 0.053) [71].

In the same study, clinicians were 3.5 times more likely to indicate confidence about their decisions when working with Visual Clot than conventional ROTEM results (OR 3.6; 95% CI 1.49–8.71; *p* = 0.005) [71]. These results are consistent with a prospective international dual-center study using Visual Clot by Rössler et al. [70]. The study showed that participants’ self-rated confidence to make a diagnostic decision was higher by 0.8 points on a 4-point Likert scale with Visual Clot (*p* < 0.001). Furthermore, a lower cognitive workload was measured with NASA-TLX scores for participants using Visual Clot, 31 vs. 52 for standard ROTEM results (*p* < 0.001) [70].

In a mixed methods study investigating physicians` perceptions for Visual Clot, 93% agreed that that VC is intuitive and easy to learn and 90% of the participants would like to have both Visual Clot and the standard ROTEM results on the same screen simultaneously when interpreting the results [72].

#### Visual blood

Visual Blood is a situation awareness–based technology designed to visualize arterial blood gas analyses (ABG) [73, 74]. The technology animates blood gas situations, showing the parameters and interactions as 3D icons flowing through a blood vessel (Fig. 5B, Additional file 3: Video). The individual parameters represented by Visual Blood are visualized according to their function and place of action. For instance, high plasma osmolarity is represented by water molecules entering the blood vessel through its wall. If a particular parameter is below a predefined threshold, corresponding icons become greyed-out, dashed, and blink. If the threshold has been exceeded, the icons appear in much higher numbers than when the parameter would be within normal range.

ABG is one of the diagnostic tools routinely used in perioperative and intensive care medicine. Its interpretation under time pressure can sometimes be challenging, especially for inexperienced team members. A user-centered visualization technology such as Visual Blood could facilitate its interpretation.

Bergauer et al., compared performance of fifty anesthetists that analyzed six ABG computer-based scenarios using Visual Blood and standard ABG printouts (total of 300 assessed cases per modality) [74]. Participants were more likely to make the correct clinical diagnosis (OR 2.16; 95% CI 1.42–3.29; *p* < 0.001). These findings show that Visual Blood can contribute to quicker initiation of a targeted therapy.

Furthermore, care providers showed higher perceived diagnostic confidence when interpreting ABG results with Visual Blood than with conventional printouts (OR 1.88; 95% CI 1.67–2.11; *p* < 0.001) [73]. Schweiger et al. [73] also showed that perceived workload was lower when study participants used Visual Blood compared with standard ABG printouts (a linear mixed model coefficient -3.2; 95% CI − 3.77 to − 2.64; *p* < 0.001).

After the study, clinicians rated statements about Visual Blood (n = 70). Sixty-five–70% agreed that it is intuitive and easy to learn [73, 74]. More than 70% of participants agreed that Visual Blood should become clinical routine [73].

### Limitations of avatar-based visualization technologies

The visualized data in these technologies is frequently preprocessed and simplified. Numeric values for vital signs are transformed into discrete visualization conditions, such as too low, normal, too high, or not measured. While this preprocessing enhances comprehension and diagnostic confidence, it reduces data precision. Numeric indicators remain essential for accurate data analysis. Furthermore, the technologies do not yet display trends, which can help healthcare providers identify slow changes over time, and which often serve as early warning signals before vital signs reach an alarm level. Therefore, avatars currently cannot replace regular monitoring but can serve as a supplement aimed at improving situation awareness. As with synthetic vision and numerical flight data, the key to success will be the optimal integration of the two technologies.

Visual Patient, Visual Clot, and Visual Blood were developed within the same research group. External validation data are needed for these systems. In addition, because the technologies are just beginning to be implemented in clinical practice, the best currently available evidence is a high-fidelity simulation study. No studies have shown patient outcome–relevant benefits yet. Only AlertWatch, Visual Patient, Blood, and Clot have been scientifically studied and validated. It is very important to study and continuously improve visualization technologies for their functionality and not just assume that they work [75].

### Future of user-centered technologies for patient monitoring

The potential applications of using avatar-based presentation of medical information seem limitless. A future vision focuses on a holistic model that combines several visual patient-monitoring technologies into an avatar; however, such a model must retain its ability to flow critical information from each part as quickly and efficiently as possible. For example, imagine zooming from the patient avatar into the lungs, heart, and blood vessels. Conversely, imagine zooming out from the patient avatar to representations of the patient’s environment and connected devices. Next steps could include patient’s installations, such as catheters, and more defined gradations or levels in the represented vital sign changes.

Avatar-based technologies can also be an easy-to-understand approach to transporting complex information from machine learning algorithms to the user. In the age of big data, machine learning and artificial intelligence can process large amounts of data and make it useful for clinicians [2]. However, clinicians are not data scientists and may have problems understanding and interpreting the results produced by machine-learning algorithms [1]. Innovative user-centered visualizations showing the necessary information to make smart decisions may help. Avatar-based patient monitoring can integrate different artificial intelligence approaches and provide complex information in a visual format; for instance, predictions of organ system failures or straightforward strategies, such as single-stream predictions of vital signs.

Avatar-based visualizations could also be presented on different digital platforms, such as a virtual or augmented reality headset or as a hologram. This would enable users to “see” the visualizations regardless of their position in the room [74].

## Conclusion

The reviewed user-centered technologies, centered around situation awareness, mark the initial foray into the realm of visualized medicine. By employing principles from psychology and cognitive neuroscience, these systems capitalize on the advantages of human sensory perception to facilitate intuitive understanding of the information presented. The ultimate objective of these technologies is to empower individuals to make improved decisions and enhance patient safety. Studying and validating visualization technologies is vital to ensure their functionality.

## Supplementary Information

**Additional file 1.** The video tutorial used in the studies to explain the Visual Patient concept and visualizations.

**Additional file 2.** The video tutorial used in the studies to explain the Visual Clot concept and visualizations.

**Additional file 3.** The video tutorial used in the studies to explain the Visual Blood concept and visualizations.

## Data Availability

The datasets used and/or analyzed in the discussed studies are available from the corresponding author on reasonable request.
